# Present-Day Vegetation Helps Quantifying Past Land Cover in Selected Regions of the Czech Republic

**DOI:** 10.1371/journal.pone.0100117

**Published:** 2014-06-17

**Authors:** Vojtěch Abraham, Veronika Oušková, Petr Kuneš

**Affiliations:** 1 Department of Botany, Faculty of Science, Charles University in Prague, Prague, Czech Republic; 2 Department of Botany, Faculty of Science, University of South Bohemia, České Budějovice, Czech Republic; 3 Nature Conservation Agency of the Czech Republic, Prague, Czech Republic; 4 Institute of Botany of the Academy of Sciences of the Czech Republic, Průhonice, Czech Republic; DOE Pacific Northwest National Laboratory, United States of America

## Abstract

The REVEALS model is a tool for recalculating pollen data into vegetation abundances on a regional scale. We explored the general effect of selected parameters by performing simulations and ascertained the best model setting for the Czech Republic using the shallowest samples from 120 fossil sites and data on actual regional vegetation (60 km radius). Vegetation proportions of 17 taxa were obtained by combining the CORINE Land Cover map with forest inventories, agricultural statistics and habitat mapping data. Our simulation shows that changing the site radius for all taxa substantially affects REVEALS estimates of taxa with heavy or light pollen grains. Decreasing the site radius has a similar effect as increasing the wind speed parameter. However, adjusting the site radius to 1 m for local taxa only (even taxa with light pollen) yields lower, more correct estimates despite their high pollen signal. Increasing the background radius does not affect the estimates significantly. Our comparison of estimates with actual vegetation in seven regions shows that the most accurate relative pollen productivity estimates (PPEs) come from Central Europe and Southern Sweden. The initial simulation and pollen data yielded unrealistic estimates for *Abies* under the default setting of the wind speed parameter (3 m/s). We therefore propose the setting of 4 m/s, which corresponds to the spring average in most regions of the Czech Republic studied. Ad hoc adjustment of PPEs with this setting improves the match 3–4-fold. We consider these values (apart from four exceptions) to be appropriate, because they are within the ranges of standard errors, so they are related to original PPEs. Setting a 1 m radius for local taxa (*Alnus*, *Salix*, Poaceae) significantly improves the match between estimates and actual vegetation. However, further adjustments to PPEs exceed the ranges of original values, so their relevance is uncertain.

## Introduction

Pollen-based quantification of past land cover is important for understanding vegetation-climate interactions and human induced changes [1]. The Landscape Reconstruction Algorithm (LRA) [2], [3] is a robust method for quantitative vegetation reconstruction, and is therefore widely used for studying Holocene sequences [4]–[9] and interglacial deposits [10]. The LRA can be used in combination with other data, for example, to estimate the spatial extent of cereal fields [7], to examine the role of different factors on long-term vegetation changes [11] or as input for climate reconstructions [12].

The necessary parameters include taxon-specific relative pollen productivity and parameters of the pollen dispersal function (such as the size of the basin, size of the region, fall speed of pollen and wind speed). Various model parameters have been shown to vary significantly among regions [13]. To provide Holocene vegetation estimates for the Czech Republic, we therefore need to examine the model parameters in light of modern pollen assemblages at fossil sites and compare them with actual vegetation composition. In the present paper, we focus only on the first step of the LRA: REgional Vegetation Estimates from Large Sites (REVEALS) [2]. This model estimates vegetation for large regions (10^6^ km^2^) based on single or multiple pollen sites and provides a baseline for the second step of the LRA: LOcal Vegetation Estimates (LOVE), which produce single-site vegetation proportions for a limited local area (few km^2^) [3].

Actual vegetation data for large areas can be compiled from various sources: forest inventories, crop statistics, land-cover information and remote sensing data such as aerial or satellite images [14]. Until now, Czech vegetation has not been reconstructed on a quantitative basis, although its components have been examined separately to solve certain partial problems such as land cover changes in the last two decades [15]. Here it is important to note that detailed qualitative overviews of vegetation in the Czech Republic are available (e.g. [16]).

The REVEALS algorithm was originally developed for large sites, as they better reflect regional vegetation than small ones (e.g., [17]). However, simulations and empirical data show that good mean estimates of regional vegetation can be attained even when many small sites are simultaneously included in a REVEALS model [2], [18]. Many studies have already successfully employed the REVEALS algorithm using the Prentice-Sugita dispersal-deposition model, but always in areas with large lakes [4]. One exception are pilot tests carried out by Sugita *et al.,* [18], who used many same-sized small bogs. This model assumes that no taxa of interest grow within the sedimentation basin. Yet, most Czech palynological records come from bogs, which differ in size and in the number of taxa of interest growing within their sedimentation basins, depending on the region. The size of the sedimentation basin influences the size of the region, hence the term “characteristic radius” [19]. Previous testing suggests that the extent of a vegetation survey (region) has little effect on model validation [20].

Apart from this dispersal-deposition consideration, it is necessary to work with correct relative pollen productivities (PPEs) for all taxa. This parameter has already been calculated for many parts of Europe, and differences in methodology (lakes vs. moss polsters) or environmental setting (climate, landscape structure) cause substantial variations [13]. Since PPEs of 13 pollen taxa from moss polsters are available for Central Bohemia [21], any methodological and environmental biases associated with taxa under study should theoretically be reduced to a minimum. However, PPEs of missing target taxa (*Picea*, *Fagus*, etc.) must be filled in with data from other areas. It has been shown that averaging different values from Europe [22] yields applicable results using the dataset from the Czech Quaternary Palynological Database [23]. These averaged PPEs, in spite of their slightly different input parameters (type and size of basin, set of taxa), assure the consistency among REVEALS estimates of past vegetation. Which PPEs are the most appropriate for actual vegetation remains uncertain, however.

The main goal of our present study was to ascertain the best REVEALS settings and adjustments of parameters for producing a reliable quantitative vegetation reconstruction. We therefore i) examined actual vegetation data for the Czech Republic, ii) tested the effects of taxa growing in peat bogs and iii) identified the best set of PPEs. We particularly addressed questions related to the effects of wind speed and the characteristic radius of regional vegetation.

### Theoretical Assumptions

The LRA [2], [3] is the inverse form of the ERV model [24]–[26]. Both methods therefore deal with space in a similar way. The whole space is divided into a sedimentation basin (R), a relevant source area of pollen (RSAP) and an area of background pollen.

No taxa producing pollen are assumed to grow in the sedimentation basin. Its radius and type are set as parameters prior to the analysis. The size of the radius can range from 0.5–1 m (e.g. in studies of moss polsters using the ERV model) to several kilometres, as in the case of some large lakes.

According to the ERV model, the area of the background pollen is defined as the source area of a certain proportion of pollen which lies beyond the RSAP and does not have an exact extent. The REVEALS model produces vegetation estimates pertaining to the area from the edge of the sedimentation basin to the maximum range of the regional vegetation (Zmax). This is an input parameter. The size of the background area is generally 10^5^ km^2^; however, the REVEALS model deals with PPEs from a much smaller area. We therefore need to validate our selection of PPEs in a REVEALS model.

PPEs are not the only parameters for translating pollen data into vegetation proportions when using the REVEALS model. As already mentioned above, the maximum range of the regional vegetation (Zmax), the radius of the sedimentation basin (R) and also parameters of the deposition function – wind speed (*u*) and fall speed of pollen (*v_g_*) – each play a significant role. We could have adhered to widely used settings of these parameters (see Methods for default settings) but decided to explore how changing these parameters affects the final results of simulations. A similar approach to testing the effect of changing radius was taken in Norway [27].

Secondly, we tested selected settings on real data and, finally, adjusted PPEs according to the dataset. Adjustment of PPEs is based on the following general assumptions: (1) The REVEALS algorithm with selected deposition function describes realistic conditions; (2) The fall speed of pollen (*v_g_*) is universally valid; (3) The given sets of pollen assemblages represent regional pollen rain; and lastly (4) Vegetation data reflect actual vegetation. If input parameters (*u*, R, Zmax) are chosen correctly, we can use the dataset to recalibrate PPEs.

### Characteristic Radius

The maximum range of the regional vegetation (Zmax) can be approximated as the characteristic radius, assuming homogenous vegetation [20]. The characteristic radius is a distance (z) from which part of the pollen loading (F*_i_*(R,z)) of taxon *i* arrives at the sedimentation basin with radius R (Equation 1). Taxon specificity is given by parameter *b_i_* that depends on how fast pollen is lost from the atmosphere. Parameter γ was set to ∼1/8, which corresponds to typical daytime conditions. The term *b_i_* is given approximately by 75 *v_g_/u*, where *v_g_* is the fall speed of the pollen and *u* is the wind velocity [19].
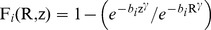
(1)


### REVEALS Model

A REVEALS estimate (V*_i_*) is the proportion of regional vegetation composition belonging to taxon *i.* It is defined for one site (*k*) as pollen counts of taxon *i* (n*_i_*) weighted by its pollen productivity (*α_i_*) and dispersal term (K*_i_*), divided by the sum of weighted pollen counts for all taxa [2]. In the case of multiple sites (Equation 2), sums of weighted pollen counts of taxon *i* from all sites are divided by the total sum of the same sums of weighted pollen counts for all taxa (*j*) at all sites (*k*) (Sugita, pers. comm.).
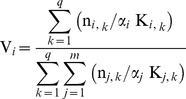
(2)


The dispersal term (K*_i_*) can be substituted by any dispersal function. We used the Prentice model (Equation 3), which considers the distance of source plants (z), dispersal properties of pollen and the type of sediment where pollen is deposited without mixing (bog model). The distance is considered from the edge of the sedimentary basin (R) to the edge of the maximum range of the regional vegetation (Zmax). A sedimentary basin is defined an area of a peat bog or lake where no plants of interest grow. However, this assumption can be violated if the size of a peat bog vegetated by target species is set to the size of the sedimentary basin. We thus propose that this fact is taken into account by considering the ecology of each taxon. For all extra-local taxa, the site radius should be set according to the size of the sedimentary basin; for local taxa, this radius should be decreased. Optimally, we would obtain a matrix of different radii for all taxa at all sites per region (R*_i,k_*); however, we do not have data about distances of local taxa from the centre of the sedimentation basin. We therefore suggest that the R of local taxa be approximated by the radius used in studies of the pollen/vegetation relationship based on pollen trapped in moss posters (i.e. 1 m).
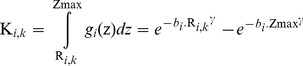
(3)


## Methods

### Characteristic Radius

We designated 70% of the pollen loading, following [19], [20], as a representative part of the major source area of pollen collected at a certain point in the canopy. Its radius is further referred to as the “characteristic radius 70”. Using the wind speed of 3 m/s and the corresponding fall speed of pollen from Table 1, we get parameter *b_i_*. We calculated distances corresponding to the “characteristic radius 70” for all taxa at all sites (120). There is an advantage to performing separate computations for all sites instead of just working with mean values: Instead of a single number, we obtain a distribution of “characteristic radii 70” that follows the distribution of site radii and reflects the dispersal properties of all taxa included in the study.

**Table 1:** Fall speed of pollen (*v_g_*), relative pollen productivity estimates (PPE) and their standard error (SE).

	*v_g_*	original PPEs			adjusted PPEs	
	(m/s)	PPE	SE	citation	*u* = 4 m/s	R_(local taxa)_ = 1 m
*Abies*	0.12	9.92	2.86	[40]	12.77	20.62
*Acer*	0.056	0.32	0.1	[41] *	0.22	0.38
*Alnus*	0.021	4.2	0.14	[89]**	4.2	6.46
*Betula*	0.024	2.42	0.2	[40]	2.62	4.31
*Carpinus*	0.042	2.106	0.0405	[48]***	0.5	0.92
Cerealia	0.06	0.0462	0.0018	[21]	0.046	0.08
*Corylus*	0.025	1.4	0.042	[89]**	1.4	2.15
*Fagus*	0.057	1.2	0.16	[41] *	1.2	1.85
*Fraxinus*	0.022	0.667	0.027	[89]**	0.18	0.28
*Picea*	0.056	0.57	0.16	[40]	0.47	0.83
*Pinus*	0.031	1.35	0.45	[40]	2	3.23
*Pla_lanc*	0.029	0.897	0.235	[90]**	0.9	1.38
Poaceae	0.035	1	0		1	1
*Quercus*	0.035	1.76	0.2	[21]	0.42	0.65
*Salix*	0.022	2.31	0.08	[91]	2.31	3.55
*Tilia*	0.032	0.8	0.029	[89]**	0.5	0.92
*Ulmus*	0.032	1.267	0.05	[89]**	6	9.23

*) in [22], **) in [7], ***) recalculated to Poaceae.

Footnotes by original PPEs indicate the source of the values. PPEs were adjusted according to the results of REVEALS model under higher wind speed (*u*) – S4MS_P and lower radius of the sedimentation basin (R) for local taxa – S4MSR_P.

### REVEALS Simulation Setting

(Table 2) We simulated the effects of increasing four parameters. Each simulation scenario has a pair scenario with control settings. In each simulation, we thus consider two effects: i) gradual changes of REVEALS estimates along the x axis and ii) changes against the control scenario. To ensure comparability between scenarios, all control simulations share the same setting at one reference point. This reference setting used the following parameters: original PPEs listed in Table 1, radius of the sedimentation basin R = 100 m and maximum range of the regional vegetation Zmax = 60 km; an even pollen assemblage of 100 pollen grains per taxon was used as the dataset. We used the Prentice model to devise the pollen dispersal-deposition function, using *b_i_* = 75 *v_g_/u*, where *v_g_* (terminal velocity) is listed in Table 1 and *u* (wind speed) equals 3 m/s.

**Table 2:** Input parameters for simulations of REVEALS vegetation estimates.

		*u*	Zmax	R (m) of the first site		R (m)	n (pollen counts)	
		(m/s)	(km)	rest of taxa	*Picea, Alnus*	second site	rest of taxa	*Picea, Alnus*
**A**	**control** *	3	60	1–1000	1–1000	–	100	100
	**scenario**	4	60	1–1000	1–1000	–	100	100
**B**	**control** *	3	1–1000	100	100	–	100	100
	**scenario**	4	1–1000	100	100	–	100	100
**C**	**control**	3	60	1–1000	1–1000	1–1000	100	100
	**scenario** *	3	60	100	100	1–1000	100	100
**D**	**control** *	3	60	100	100	–	100	100–1100
	**scenario**	3	60	100	1	–	100	100–1100

*) includes calculation of standard errors.

All calculations were performed with original PPEs (Table 1) using the Prentice model.

We asked the following questions: Simulation A – What is the effect of increasing R on REVEALS vegetation estimates of each taxon? How is it influenced by higher wind speed? Simulation B – What is the effect of increasing Zmax on REVEALS vegetation estimates of each taxon? How is it influenced by higher wind speed? Simulation C – How do REVEALS vegetation estimates change when we calculate them for two sites differing in size? Simulation D – Can approximation of R by the moss polster size of 1 m for local taxa improve REVEALS vegetation estimates when local taxa have higher pollen proportions than other taxa? We expected the results to be influenced by PPEs and fall speed of pollen. We therefore selected *Picea* and *Alnus* (see their values in Table 1) as local taxa in simulation D. This combination represents the tree layer of the wetland community *Thelypterido palustris-Alnetum glutinosae* [28].

### Pollen Data

**Figure 1:**
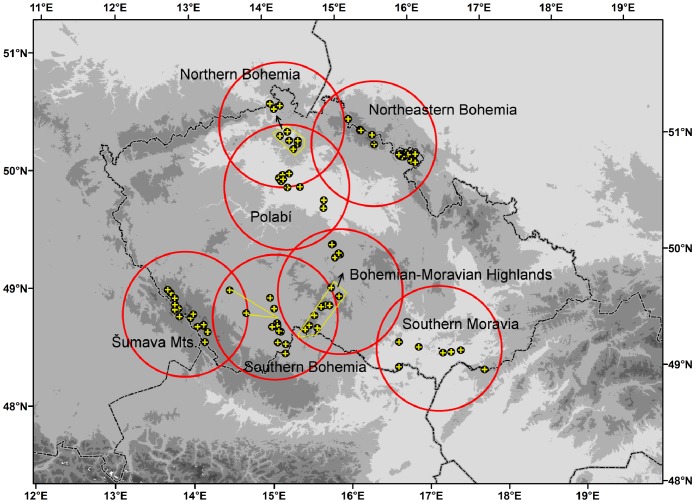
Map of the Czech Republic with pollen sites (yellow crosses) and assigned regions (red circles). Northern Bohemia [49]–[53], Northeastern Bohemia [49], [54]–[63], Polabí [64]–[67], Southern Bohemia [68]–[73], Southern Moravia [74]–[79], Bohemian-Moravian Highland [80]–[85], Šumava Mts. [86]–[88]. Yellow lines and arrows show the allocation of sites to regions in cases when sites are situated in two overlapping regions.

We used the shallowest sample from every core in the Czech Quaternary Pollen Database (PALYCZ, accessed 01.07.2013). However, these samples were not collected in the same year as the vegetation data (see Table S1), but within the last 40 years, and not all of them have exactly 0 cm depth. Still, we assume that they are the closest representation of recent pollen deposition. Centroids of regional circles were placed visually around the spatial clusters of the sites. When two regions overlapped, certain sites fell into both of them at the same time. In these cases, the region was assigned according to the environmental conditions surrounding the core (vegetation, altitude, climate, etc.) (Figure 1). The pollen sum of selected taxa was limited to 100 pollen grains per sample, but only seven sites had less than 200 grains per sample; the average pollen sum of all 120 sites is 542 grains per sample (Table S1). Names of genera, except *Pinus*, used to denote selected pollen types refer to all species within the given genera. The name *Pinus* refers only to species belonging to the subgenus *Pinus* (diploxylon pines). *Plantago lanceolata* is the only pollen taxon defined at the species level. Poaceae encompass all wild grasses, and Cerealia comprise the genera *Triticum*, *Hordeum* and *Avena*; if distinguished, pollen of *Secale* and *Zea* was excluded.

### Data Sources of Actual Vegetation

To establish the proportions of all target taxa in actual vegetation (Table 1) and the size of each regional circle (Figure 1), we extracted data from various sources. Distribution and abundance information is available for two groups of taxa. Data on the first group (crops and trees) can be relatively easily obtained because its biomass is ascertained periodically, as they directly benefit mankind. Data on the second group of taxa (Poaceae, *Plantago lanceolata* and *Corylus*) are available thanks to the habitat mapping project coordinated by the Nature Conservation Agency of the Czech Republic, which was initiated for delimiting Natura 2000 sites and is continually updated for the purpose of reporting under the Habitats Directive (92/43/EEC). We used the habitat mapping layer (Nature Conservation Agency of the Czech Republic, unpublished data) – a large dataset containing spatial and tabular data from habitat surveys (species presence or abundance) – to estimate the proportion of taxa in the second group. The first results (number of segments and area of every habitat) have already been published [29].

We used several data sources to obtain the most reliable data on regional vegetation cover. The CORINE Land Cover (CLC) map from 2006 [30] served as a spatial basis for most of the area. Areas under farm crops from 2006 [31] in the resolution of administrative regions (average area 5,637 km^2^) were included in CLC class 211 (Arable fields). Forest inventory data from 2006 [32] in the resolution of municipalities with extended competence (average area 383 km^2^) were joined with CLC classes 311, 312 and 313 (coniferous, deciduous and mixed forest). Areas of forested and arable land provided by numerical and spatial data (statistics and CLC) were compared at the corresponding resolution.

### Processing of Habitat Mapping Data

We combined the CLC map with habitat mapping data (see below) to obtain average abundances of Poaceae, *Corylus* and *Plantago lanceolata*. The following reasons made us select these taxa: Poaceae are the key taxon of open landscapes, *Corylus* became subdominant in pollen assemblages during the Early Holocene, and *Plantago lanceolata* is classified as an anthropogenic indicator [33]. These taxa allow us to reconstruct landscape openness, Early Holocene vegetation and the magnitude of human impact. The habitat mapping layer consists of two datasets: (i) a map of natural habitats covering 20% of the Czech Republic obtained by a field survey carried out between 2000 and 2011; and (ii) the presence or abundance of taxa estimated in some segments (0.4% of the total area for Poaceae, *Corylus* and *Plantago lanceolata*) collected between 2008 and 2011. The whole map of habitats was intersected with the CLC map. Abundance data were averaged and extrapolated to all habitats and CLC classes.

The details of the method of habitat mapping are described elsewhere [29]; however, it is helpful to mention its most important aspects. Recorded taxa include vegetation dominants and taxa of interest for nature conservation. In segments with habitats categorized as “natural”, abundances of diagnostic or typical species were recorded using the Braun-Blanquet scale. In segments with transitional or human-influenced biotopes, only the presence of target species was recorded. The Braun-Blanquet scale was converted into percentages according to the following key: “r”–0.1%; “+”–0.5%; “1”–3%; “2”–15%; “3”–37.5%; “4”–62.5; “5”–87.5%. The total cover of segments had to be standardized due to the presence of multiple vegetation layers (more than one species recorded as “5”). If the total cover of a segment was less than half the standard value, the record was treated as a mere presence. The standard total cover was obtained from [34], [35].

The extrapolated cover of *Corylus*, Poaceae and *Plantago lanceolata* was calculated by bootstrapping from random resampling from all segments with replacement. We then calculated the average percentage for segments containing abundance data of the habitat/CLC class and multiplied it by its proportional presence in all segments. Bootstrap calculations (N = 5000) of these weighted means permitted estimation of variances for each habitat/CLC class and standard deviation in each region (see Table S2, S3, S4 in Supporting Information). Due to the focus of vegetation mapping on natural and semi-natural biotopes, the surface of some CLC classes suitable for vegetation mapping was estimated as follows: 35% – discontinuous urban fabric, industrial or commercial units (121, 112), 10% – forests (311, 312, 313) and 5% – arable land (211). Herb taxa (Poaceae and *Plantago lanceolata*) were considered only in non-forest and shrub vegetation (excl. T, K, X9, X8 sensu [36]).

The layer was intersected with circles representing regions. Some of the circles extended beyond the borders of the Czech Republic. We therefore extrapolated the vegetation composition in parts of the circles within the country to parts of the circles overlapping into neighbouring countries. Similarly, we assumed regional proportions of bare land and areas vegetated by plants beyond our interest to be zero. We converted absolute plant abundances into vegetation proportions to compare them with vegetation estimates.

### Data Analysis

**Figure 2:**
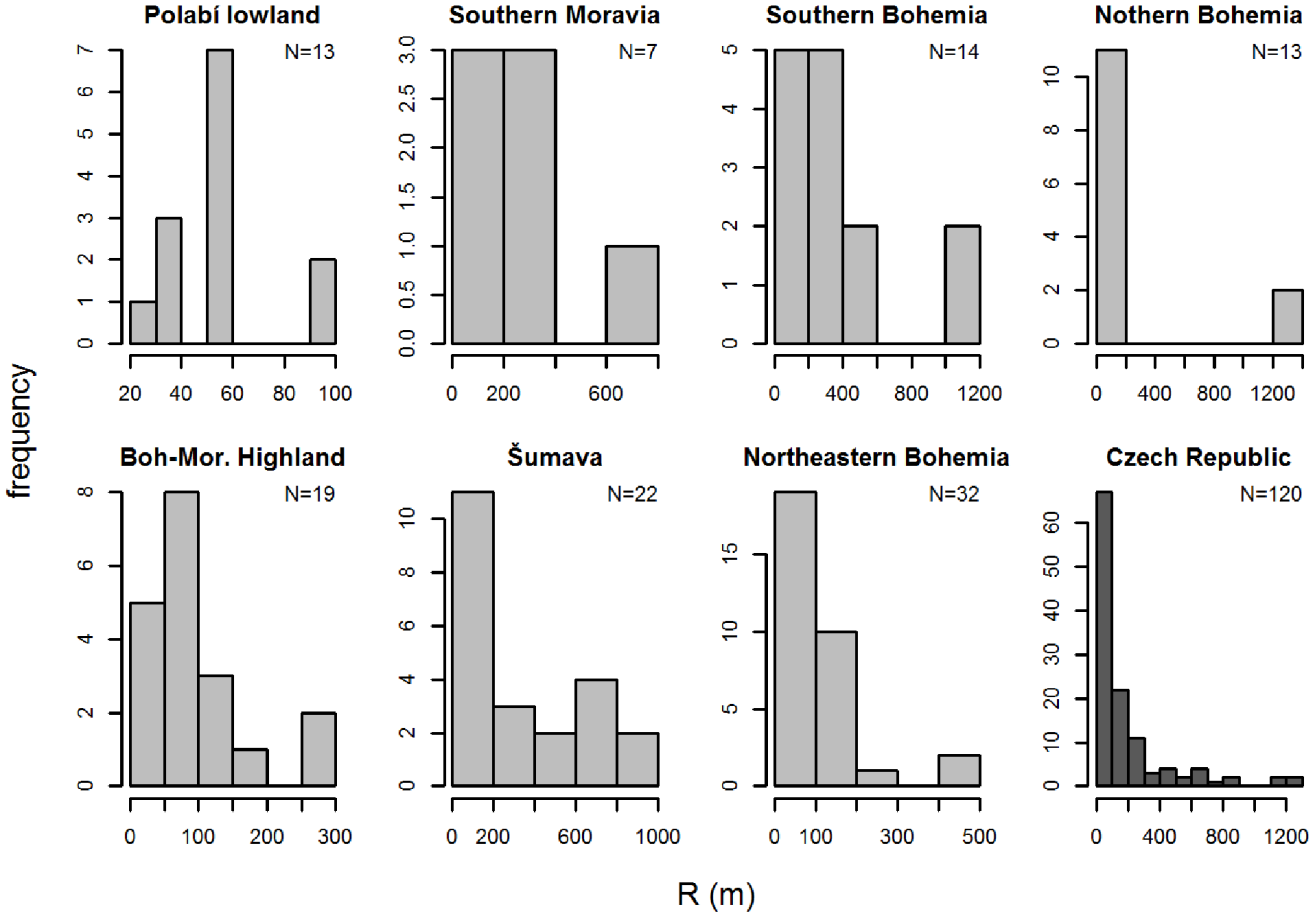
Histograms of sizes of sedimentation basins in different regions. The number of sites included is indicated at top right.

REVEALS estimates were calculated by the REVEALS.v4.2.2.Tallinn.wks.exe binary (Sugita unpublished) and by a script written in R [37] (Text S1). Unlike our script, the original programme calculates standard errors, but offers only limited parameter settings. These default settings together with our initial parameters include: Prentice’s [38] model as the dispersal function using *b_i_* = 75 *v_g_*/*u*, the fall speed of pollen (*v_g_*) extracted from previously published works [39]–[41] and wind speed (*u* = 3 m/s). The radius of the sedimentation basin (R) was set to the size of the peatbog, a parameter extracted from the Czech Quaternary Palynological Database (Figure 2, Table S1). The maximum range of the regional vegetation Zmax was set 60 km.

We selected PPEs during preliminary REVEALS calculations. Assuming environmental similarities, we first picked PPEs for all available taxa in Central Bohemia; PPEs for missing taxa were completed from other studies with the following priority Swiss Plateau, Swiss Jura and Southern Sweden. PPEs of taxa not matching vegetation estimates were substituted by other values until we reached the best set of PPEs. A change of one PPE value shifts the estimates of all taxa; however, rare taxa have a smaller effect than abundant ones, so we began by evaluating abundant taxa. We first assessed the goodness of fit visually and then calculated two kinds of dissimilarities, hereafter referred to as “dissimilarity A” and “dissimilarity B”. The two dissimilarity indices highlight differences of REVEALS model from pollen proportions and also differences among alternative scenarios (representing expected improvements over widely used settings). Dissimilarity A was calculated by subtracting estimates (or pollen proportions) from actual vegetation values and summing the results of this subtraction for each taxon across all regions. As regards dissimilarity B, the results obtained by the previous subtraction (calculated for dissimilarity A) were squared, summed together for each taxon and divided by the sum of squared residuals from a linear regression calculated between the two variables for each taxon. To keep dissimilarity B close to 1, the linear regression line should be close to the line of best fit (one-to-one line). Another particularity of dissimilarity B is that it can be very high when both variables are linearly related, albeit far from the line of best fit.

We hypothesize that the results of our regional vegetation reconstruction in the study area can be biased by individuals growing in the sedimentation basin and by wind speed. Wind speed (*u*), the radius of the sedimentation basin (R) and PPEs were adjusted in a three-step process considering alternative scenarios. After every step, we assessed the goodness of fit between REVEALS estimates and actual vegetation proportions. First, we ran the REVEALS model with two different wind speeds: 3 m/s and 4 m/s (scenarios S3MS and S4MS, respectively). Average seasonal wind velocity in spring varies from 2.5 m/s in lowlands to 4.5 m/s in mountains [42]. In this first step, it was assumed that no taxa of interest grow in the sedimentation basin, whose size corresponds to the default setting.

In the second step, we added the wind speed setting which better matched actual vegetation proportions to the alternative setting representing the radius of the sedimentation basin. The presence of local taxa was estimated by comparing pollen percentages, general wetland vegetation of the regions and the ecology of species corresponding to our pollen taxa. Approximation using the size of moss polster sites (radius of the sedimentation basin R = 1 m) was applied to *Alnus*, *Salix* and Poaceae at all sites within the following regions: Southern Moravia, Southern Bohemia, the Polabí lowland and Northern Bohemia; *Alnus* and Poaceae were also considered local in the Bohemian-Moravian Highland.

Finally, the two scenarios with the lowest sums of both dissimilarities were used for the adjustment of PPEs. As we approached the optimal set of adjusted PPEs, dissimilarity A tended to decrease to 0, while dissimilarity B decreased towards 1. PPE values of mismatching taxa were adjusted until the best match was obtained. If the PPE of Poaceae (reference taxon = 1) needed to be adjusted, all values were adjusted accordingly to keep the value of Poaceae at 1. This was done to retain comparability with other studies.

To test the robustness of REVEALS vegetation estimates, we applied a leave-one-site-out approach. Following [22], we calculated non-parametric Spearman rank-order correlation coefficients and their statistical significance for the relationship between estimates for all sites (default scenario) and estimates with one site omitted. We tested the null hypothesis (H0) that there is no association between the two types of estimates and used a two-tailed test with the significance level of p = 0.01. Additionally, we calculated scores of Principal Component Analysis (without transformation) for all leave-one-site-out samples and their corresponding estimates for all sites. For each region, we compared the variability of all leave-one-site-out samples and also their distances from the default scenario. We expected the site radius to have a substantial influence, so we plotted each leave-one-site-out sample as a symbol whose size indicates the radius of the corresponding site.

## Results

### Characteristic Radius

**Figure 3:**
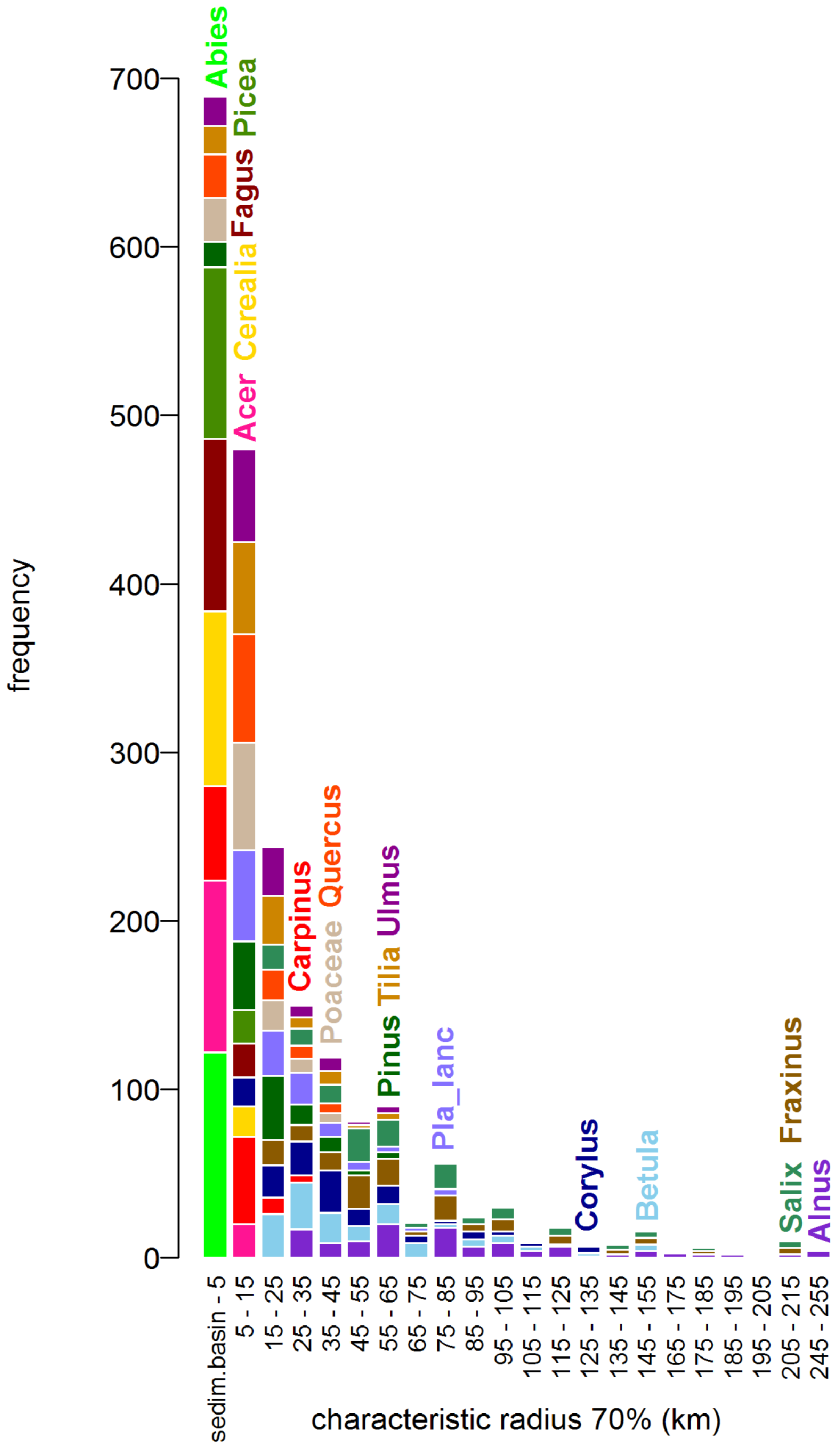
Histogram of “characteristic radii 70” (km) for all pollen taxa (17) at all sites (120). Note the different distribution of taxa with light pollen grains (e.g. *Alnus –* purple) and heavy pollen grains (e.g. *Abies –* light green). The wide distribution is given by the variability of the radii (see Fig. 2). The name of each taxon is placed at the maximum distance given by the maximum sedimentation basin (1,262 m).

(Figure 3) The maximum “characteristic radius 70” refers to the distance of approximately 250 km pertaining to all taxa at all sites. This large distance is given by the size of the largest sedimentation basin (1,262 m) and by taxa with light pollen (*Alnus*, *Fraxinus* and *Salix*). However, most of the “characteristic radii 70” are accumulated within the first 60–80 km. Within this distance fall also the maximum characteristic radii of taxa with medium-weight to heavy pollen grains. The reason behind this result is that the distribution of site radii is skewed towards smaller radii, with quartiles ranging from 30 to 250 m (Figure 2).

### REVEALS Simulation

**Figure 4:**
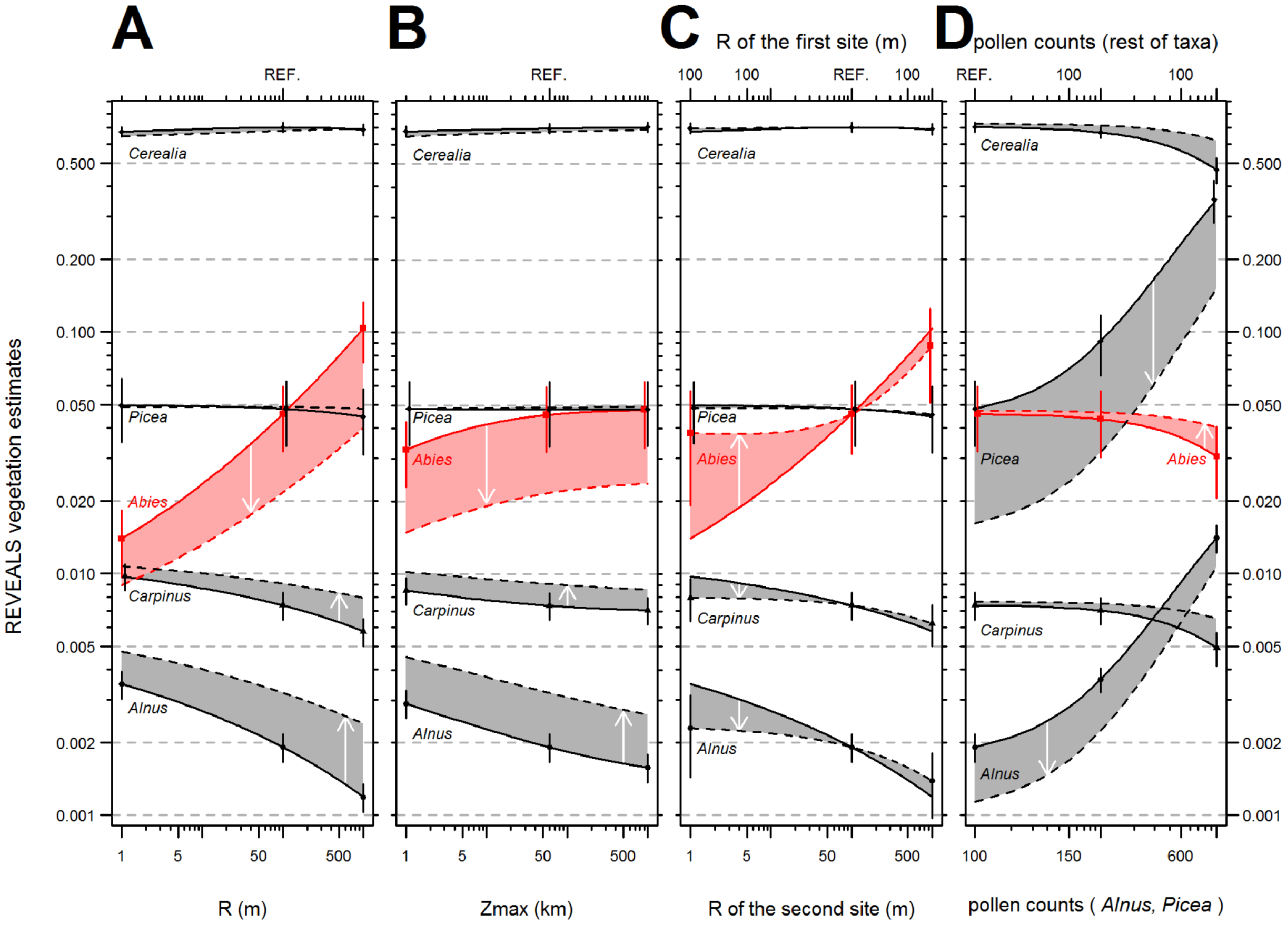
Simulation of REVEALS estimates (proportion in log scale). *Alnus-Capinus*, *Picea*-Cerealia and *Abies* delimit three groups of terminal velocities of pollen: slow, medium and fast, which also represent the pattern of the rest of taxa not plotted here. Arrows show the change of estimates from control (full line) to scenario (dashed line); for details, see Table 2. (A) Simulation A: Increasing radius of the sedimentation basin under two wind speed velocities (B) Simulation B: Increasing radius of the area of the background pollen under two wind speed velocities. (C) Simulation C: Fixed versus increasing radius of the sedimentation basin of the second site. (D) Simulation D: Increasing pollen counts of *Alnus* and *Picea* under two radii of the sedimentation basin. The reference setting common to all control simulations is marked on the secondary horizontal axis by “REF”.

(Figure 4) Pollen productivity and the dispersal term are inversely proportional to REVEALS estimates. Hence, the highest REVEALS estimates are those for Cerealia, which have the lowest PPE. However, *Abies*, the taxon with the highest PPE, does not have the lowest REVEALS estimates because it has a low dispersal term. The first two simulations (Figure 4A, 4B) show how parameters of the dispersal term – wind speed (*u*), fall speed (*v_g_*), radius of the sedimentation basin (R) and maximum range of the regional vegetation (Zmax) – influence REVELS vegetation estimates. Taxa within each simulation show a similar pattern to taxa with similar fall speed of pollen. *Alnus-Capinus*, *Picea*-Cerealia and *Abies* delimit three groups of taxa corresponding to three ranges of terminal velocities of pollen: slow (0.021–0.042 m/s), medium (0.056–0.06 m/s) and fast (0.12 m/s). All remaining taxa fall within one of these ranges (Table 1).

Light pollen grains show a significantly decreasing trend. Medium-weight pollen grains indicate no trend or one that is only slightly decreasing or increasing. Heavy pollen grains of *Abies* show a significantly increasing trend. Higher wind speed increases REVEALS estimates of light pollen grains, decreases estimates of heavy pollen grains and has no significant influence on medium-weight pollen grains. In general, decreasing the radius of the sedimentation basin has a similar effect as increasing the wind speed parameter from 3 to 4 m/s.

Within the reasonable Zmax ranges of ca. 10–500 km, the increasing maximum range of the regional vegetation has very little effect on REVEALS estimates for all taxa.

When a combination of two differently sized sites is used (Figure 4C), REVEALS estimates of *Abies* and light-pollen taxa are similar to REVEALS estimates calculated for the larger site only (or two sites the size of the larger one). Our comparison of standard errors from the first and third simulation shows that combining two sites differing in size increases standard errors. The standard errors grow with the size difference in taxa on which the size of the site has a notable effect (e.g. *Abies* and, to a lesser extent, light-pollen taxa).

Decreasing the site radius of *Picea* and *Alnus* (100 m → 1 m) decreases the REVEALS estimates of these taxa (Figure 4D). This effect is stronger for *Picea* than for *Alnus* because a pollen assemblage with a site radius of 1 m and 210 more *Picea* grains or more 70 *Alnus* grains than the rest of the taxa produces similar REVEALS vegetation estimates as an assemblage with a 100 m radius and equal pollen counts for all taxa. It is important to note the difference against the first simulation; in other words, when the site radius decreases for all taxa, REVEALS estimates of *Alnus* increase.

It is necessary to note the log scale of the vertical axis. Changes in the vegetation estimates of *Alnus* (Figure A–C) are actually very low (<0.5%). Similarly, however, although the decreases in control simulation D of Cerealia, *Abies* and *Carpinus* seem similar; Cerealia exhibit the highest absolute decline (70 → 47%).

### Actual Vegetation Cover Derived from CLC and Habitat Mapping

**Figure 5:**
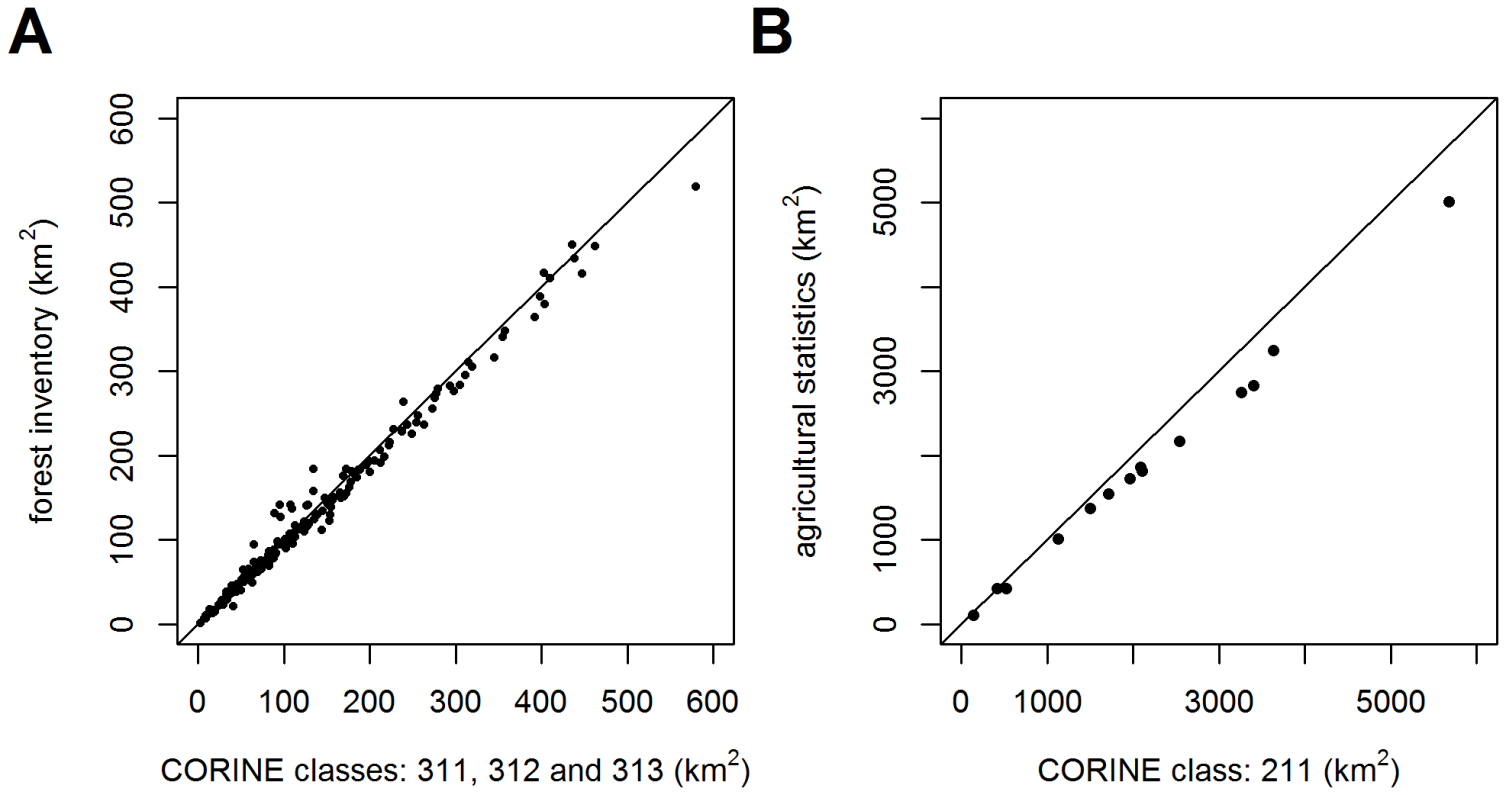
Comparison of the area derived from the CLC map [30] and numerical data sources. (A) Forest area within municipalities with extended competence [32]. (B) Arable land within regions [31].

**Figure 6:**
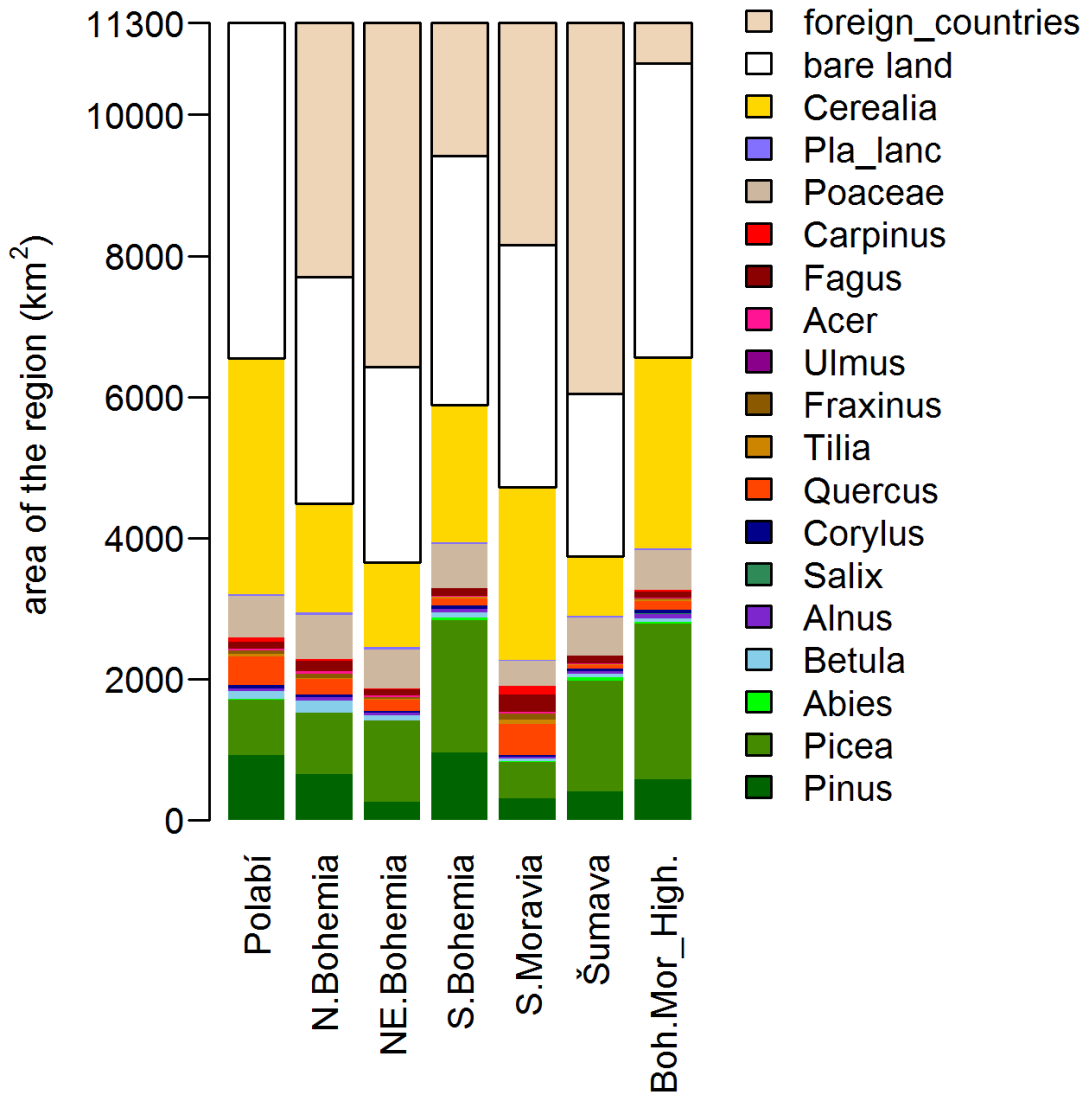
Vegetation cover in selected regions.

(Figure 5, 6) Areas of arable land and forests derived from two different sources yield similar results and are thus combinable (Figure 5). There is a slight trend towards overestimating forest and arable land in CLC mapping with increasing size of the municipality or region.

The selected taxa cover 56% of the area of the Czech Republic, dominants being Cerealia, *Picea*, Poaceae and *Pinus*; the remaining taxa never reach 2%. The taxa reach similar proportions in all regions (Figure 6). The selected taxa cover only 35–57% of the surface of the regions studied (60 km radius circles). The remainder of the circles is bare land, land covered by other taxa or land outside the Czech borders. Standard deviations of regional proportion of Poaceae, *Plantago lanceolata* and *Corylus* turned out to be lower than the plotting limit (<0.1%), so they are only listed in Table S4.

### Pollen-based Estimated Vegetation by REVEALS Model

**Figure 7:**
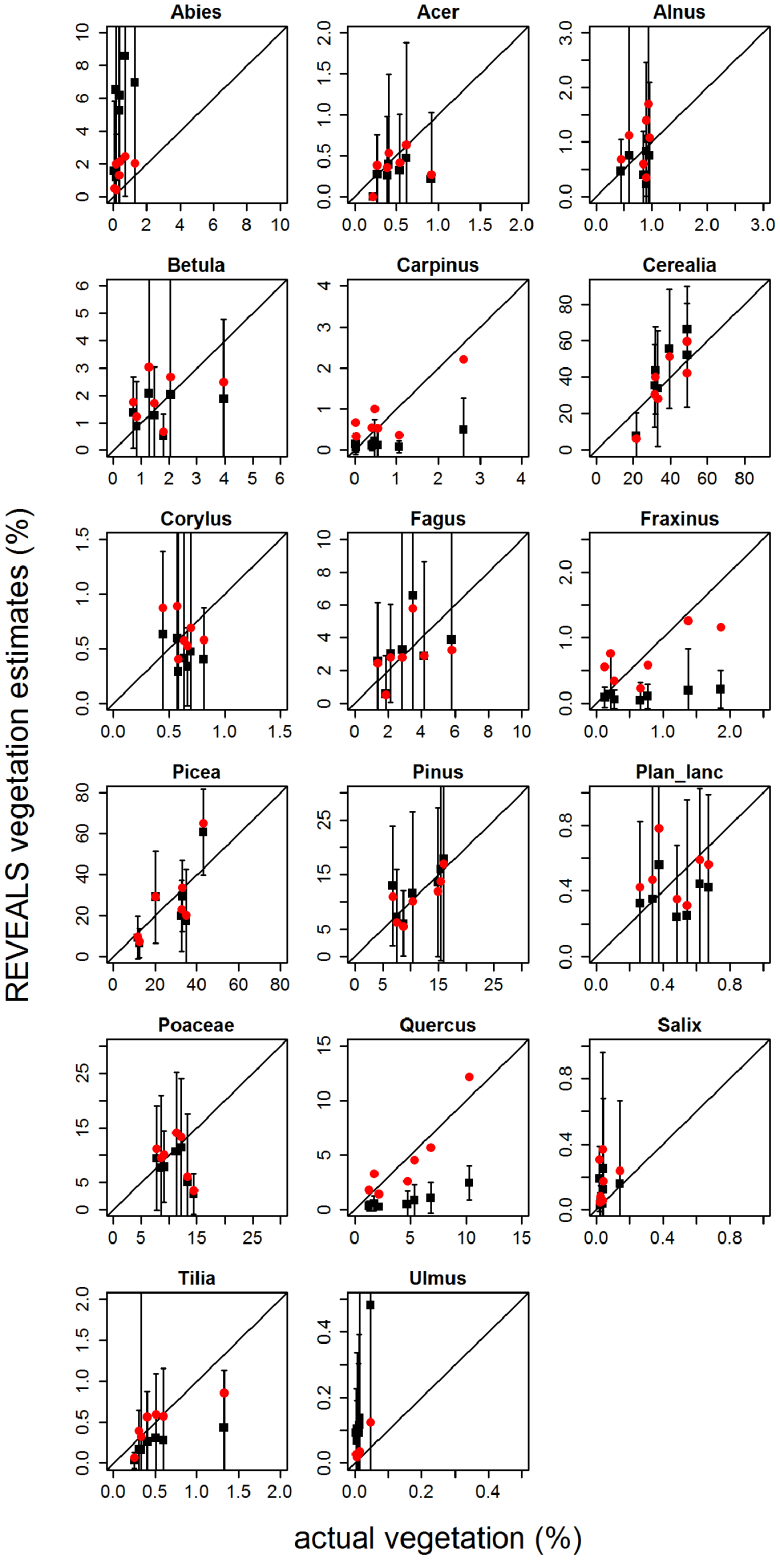
Comparison of actual regional vegetation with REVEALS estimates. Settings: original PPEs under wind speed of 3 m/s - S3MS (black squares, confidence intervals show their standard errors); adjusted PPEs under wind speed of 4 m/s - S4MS_P (red dots). Both settings deal with original sizes of the sedimentation basin. The diagonal line shows the position of the optimal fit of the model to expected values.

**Figure 8:**
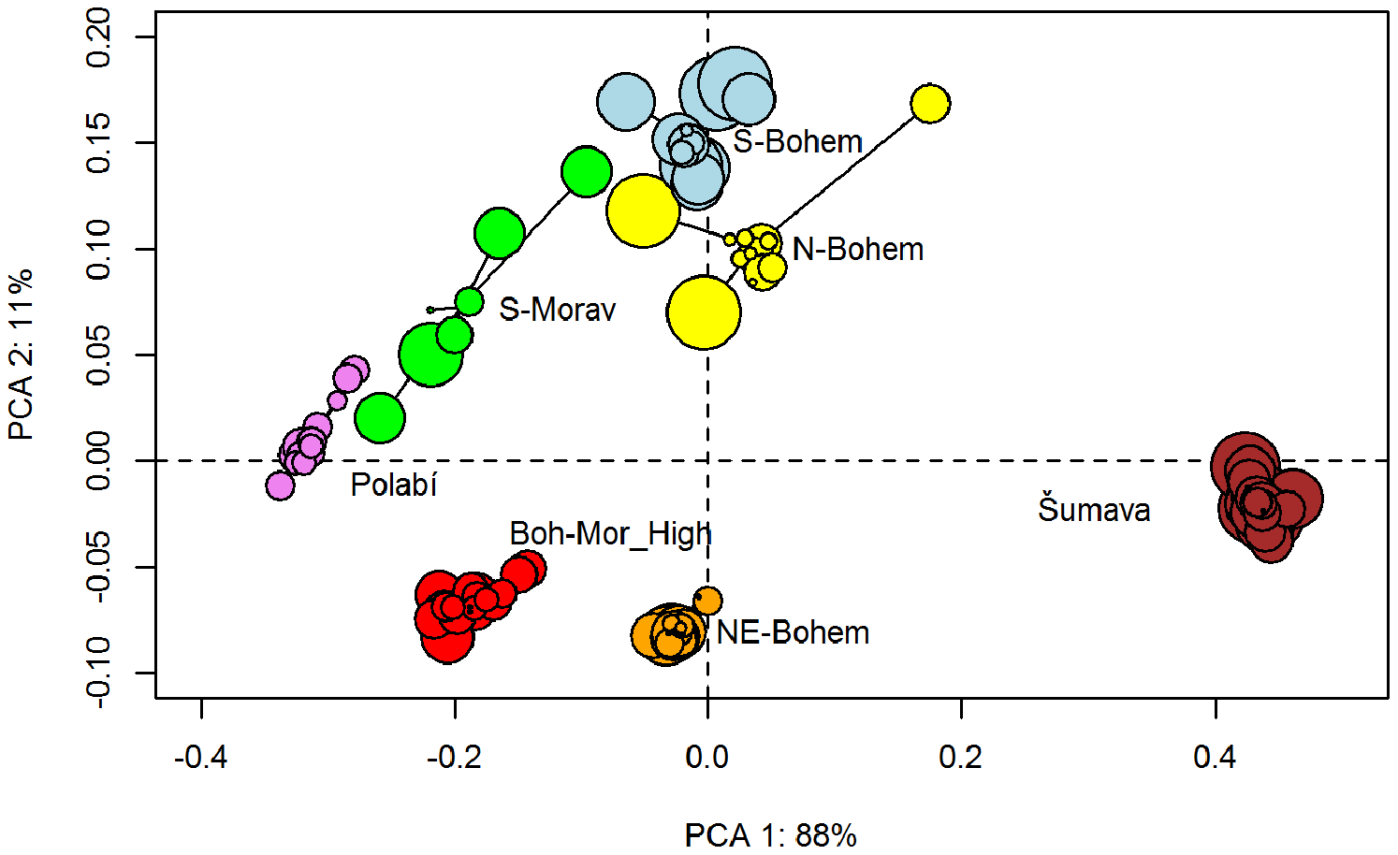
Principal Component Analysis of REVEALS estimates. Colours cluster all leave-one-site-out runs to their corresponding regions. The size of each sample reflects the size of the site which was left out.

(Figure 7, 8) Table 3 shows both measures of dissimilarity between REVEALS estimates and actual vegetation. Their sums (0.32–1.6 and 64–957) are several times lower than in the case of pollen proportions (7.31 and 4875). According to both dissimilarities, scenario S4MS better matches actual vegetation than the original setting (S3MS). Considering the taxa individually, *Abies*, Poaceae and Cerealia show the best improvement in these three scenarios; on the other hand, the match of *Pinus* and *Picea* deteriorates. The setting from scenario S4MS was used in cases of local taxa with small radii (Poaceae, *Salix*, *Alnus*).

**Table 3:** Two measures of dissimilarity between actual vegetation and REVEALS estimates or pollen proportion.

	dissimilarity A						dissimilarity B					
	sum of simple differences						sum of quadratic differences/sum of quadratic residuals					
		step 1		step 2	step 3			step 1		step 2	step 3	
	pollen	S3MS	S4MS	S4MSR	S4MS_P	S4MSR_P	pollen	S3MS	S4MS	S4MSR	S4MS_P	S4MSR_P
Abies	−0.11	−0.33	−0.12	−0.1	−0.08	−0.09	3.4	6.7	5.4	9.7	4.3	4.3
Acer	0.03	0.01	0.01	0.01	0.01	0.01	59.1	5.1	5.3	1.8	2.2	2.5
Alnus	−0.5	0.01	−0.02	0	−0.01	0	4.3	1.6	1.3	1.2	1.2	1.1
Betula	−0.53	0.02	−0.03	−0.05	−0.01	−0.02	6.3	3.8	2.2	2.2	2.2	2.1
Carpinus	0.01	0.04	0.04	0	–0.01	0	7.4	152.9	106.6	2.4	2.0	2.3
Cerealia	2.43	−0.38	−0.11	−0.22	−0.03	0.03	4626.4	2.4	1.5	1.5	1.3	1.3
Corylus	−0.08	0.01	0	−0.01	0.00	−0.01	4.3	6.7	2.4	1.5	2.6	2.7
Fagus	0.01	−0.01	0	−0.04	0.01	−0.01	1.4	1.2	1.3	1.1	1.4	1.3
Fraxinus	0.03	0.04	0.04	0	0.00	0	41.7	382.5	123.3	2.0	3.0	2.2
Picea	1.01	0.15	0.25	0.2	0.00	0.06	10.8	1.1	1.1	1.4	1.1	1.1
Pinus	−1.86	−0.06	−0.36	−0.08	0.04	−0.01	31.3	1.2	3.1	1.1	1.2	1.0
Pla_lanc	−0.03	0.01	0	−0.01	0.00	−0.01	2.5	3.5	1.8	1.4	1.9	2.0
Poaceae	−0.44	0.22	0.07	0.33	0.09	0.09	21.9	5.1	3.1	107.4	3.0	4.4
Quercus	0.12	0.26	0.24	−0.03	0.01	−0.03	19.7	274.7	131.8	1.1	1.0	1.2
Salix	−0.06	−0.01	−0.01	−0.01	−0.01	−0.01	3.5	2.0	2.5	2.9	2.4	2.6
Tilia	0.01	0.02	0.02	0.01	0.00	0.01	4.5	42.0	15.7	4.7	2.5	3.1
Ulmus	−0.03	−0.01	−0.01	0	0.00	0	26.8	64.9	63.3	24.6	31.3	34.8
sum of absolute values	**7.31**	**1.6**	**1.33**	**1.09**	**0.32**	**0.38**	**4875.1**	**957.2**	**471.7**	**168.0**	**64.5**	**70.2**

Note the improvement of sums against previous steps. Abbreviations code the REVEALS settings: S - wind speed, R - radius of local taxa decreased to 1 m, _P - pollen adjusted.

Scenario S4MSR generally yields brings estimates which are closer to actual vegetation because the sum of both dissimilarities is lower than in scenario S4MS; individually, however, Poaceae and Cerealia perform worse. The lowest dissimilarities appear in scenarios S4MS and S4MSR, so both settings (i.e. wind speed of 4 m/s and wind speed of 4m/s together with the reduced radius for local taxa) are used to adjust the PPEs.

Initial PPEs and their geographical origins are summarized in Table 1, which also shows adjusted values for both alternative scenarios. Considering scenario S4MS, adjusted PPEs exceed the ranges of previously published values for *Fraxinus* 0.18, *Ulmus* 6, *Quercus* 0.42 and *Carpinus* 0.5. In the cases of Cerealia (0.046), *Pinus* (2), *Abies* (12.77), *Tilia* (0.5), *Picea* (0.47), *Acer* (0.22), *Betula* (2.62) and *Plantago lanceolata* (0.9), adjusted PPEs stay in the ranges of standard errors or close to them. Most of adjusted PPEs using settings from scenario S4MSR are higher than the initial PPEs and out of the range of their standard errors. This is because adjustment decreased the PPE of Poaceae. The sums of the two dissimilarities for both scenarios of adjusted PPEs (S4MS_P, S4MSR_P) are very similar (0.32 vs. 0.38 and from 64.5 vs. 70.2, respectively; Table 3).

All REVEALS estimates calculated by the leave-one-site-out approach across all regions are significantly correlated with corresponding values calculated for all sites (p<0.01). We therefore reject the null hypothesis. The PCA (Figure 8) shows that the highest variability among leave-one-site-out estimates lies within Northern Bohemia, Southern Bohemia and Southern Moravia. Moreover, the variability within Northern Bohemia is caused by large sites. In other words, REVEALS estimates deviate far from the mean when a large site is excluded.

## Discussion

### Accuracy of Actual Vegetation Data

#### Agricultural statistical data and forest inventories

Areas obtained from CLC classes (211, 311, 312 and 313) tend to be overestimated when compared with areas derived from forest and agricultural statistics. Such discrepancies are caused by small-scale owners of forests and arable land, who are not required to maintain forest inventories or to provide data for agricultural statistics. This lack of accuracy becomes apparent in larger areas because of accumulated noise. Another reason behind the overestimation of forest CLC areas is that military zones, although abandoned and overgrown by forests, are registered as non-forest areas. Even though there is a tendency to overestimate forest CLC areas, some municipalities have underestimated them. Such municipalities are located within regions with high forest regeneration, since young forests do not show up as forested areas in remote sensing. Fortunately, these kinds of errors do not on average exceed 6% in the area considered here. Forestry data on taxa deemed marginal for timber production may lack accuracy and should therefore be considered unreliable, particularly in cases of rare tree taxa with actual vegetation data below 0.1% (*Carpinus* in two regions, *Ulmus* and *Salix* in six regions, *Abies* in one region).

#### Habitat mapping

This third source of vegetation data was used for extrapolation. Habitat mapping covers a high number of independent observations over a relatively large area; however, the number of records for Poaceae, *Corylus* and *Plantago lanceolata* differs (Table S2). These variations are due to different frequencies of these three taxa in the vegetation, but we must point out certain biases introduced by methodological aspects of the mapping. The taxon Poaceae consists of dominants or diagnostic species of many important habitats and plant communities (e.g. *Corynephorus canescens*, *Molinia caerulea*, *Arrhenatherum elatius* and *Bromus erectus*). These species were recorded relatively well, at least when it comes to their presence. *Corylus avellana* is favoured by nature protection, so it was a well recorded taxon in all habitats where it was present and not only in habitats where its recording was compulsory (K3, L3.1, L3.2, L3.3, L8.2 and S1.5 sensu [36]). On the other hand, mapping of *Plantago lanceolata* was the least accurate because it is utterly uninteresting from the standpoint of nature conservation and thus its recording was compulsory only in a few habitats (T1.3, T3.5A, T3.5B and T5.5) [43]. Its mean values are nevertheless based on 7,363 segments with recorded abundances and 21,319 segments with recorded presence, which we consider sufficient.

### Reveals

Generally, estimates of the REVEALS model from all scenarios are several times closer to vegetation data than mere pollen proportions (see Table 3 and Figure 7). However, *Abies* and *Quercus* matches were poorer worse when using the REVEALS model with standard settings (*u* = 3 m/s) than simple pollen proportions.

### Maximum Range of the Regional Vegetation (Zmax)

The general grain of the landscape mosaic in Central Europe is sufficiently fine to fulfill the assumption of vegetation homogeneity for the area of the background pollen [19]. Landscape heterogeneity can matter if we get for a comparison with REVEALS estimates different regional vegetation at different radiuses, for example if there is an altitudinal gradient of vegetation. The centre of the Šumava region is situated in forested mountains, but the peripheral part of the circle reaches lowlands with a cultural landscape. On the other hand, Zmax (as a one of the REVEALS parameters) has little effect on vegetation estimates in accordance with Hellman *et al* [20]. So in practice, when comparing regional vegetation estimates and regional vegetation data, setting the Zmax parameter is more important for the vegetation survey. Heterogeneous mosaics are inevitable in real landscapes, but the theoretical size of the region can be at least approximated using characteristic radius concept. Most “characteristic radii 70” of our set of taxa and set of sites are smaller than 60 km; i.e. the length we set as the maximum range of the regional vegetation.

### Wind Speed (u)

The REVEALS settings with the wind speed parameter of 4 m/s decreased the sums of dissimilarity A and dissimilarity B compared to the default settings. The lower sum of dissimilarity A can also be attributed to the decrease in the dissimilarity of *Abies*. Our simulation shows that lower REVEALS estimates for *Abies* can be attained using the same pollen counts provided that either the sedimentation basin is smaller or wind speed is faster.

To decrease the mismatch of *Abies* caused by the default settings, we could have also decreased the sedimentation radius, either for *Abies* or for all taxa. Although *Abies* can grow in wet subtypes of phytosociological associations [44], it is not a typical tree of wetlands. Decreasing the radius would entail neglecting the size of the sedimentation basin as an important theoretical concept of pollen analysis.

We also tried to keep default setting (S3MS) and adjusted the PPE for *Abies* in one of the preliminary analyses, which resulted in a PPE of *Abies* higher than 20. However, such a high PPE for *Abies* is well out of any range of PPEs published so far and does not seem very realistic.

We thus decided to correct the mismatch of *Abies* by setting the wind speed parameter to 4 m/s. This finding corresponds to observations of pollen trapping, which show that heavy pollen grains fall closer to their trees and that only a small part of them is able to reach air currents high enough above the canopy and thus contribute to the regional component of pollen rain [45].

Moreover, this value falls within the theoretical range of wind speeds blowing above the canopy [46]. In most of the regions considered, the average wind speed is approximately 4 m/s. Winds in the Polabí lowland and in South Bohemia are somewhat slower [42]. The wind speed of 4 m/s corresponds better to pollen and regional vegetation data in this study, especially because *Abies* is included in the set of taxa.

### Combining Different-sized Sites for REVEALS Estimates

A correctly set radius of the sedimentation basin becomes important when *Abies* or light-pollen taxa are included in the dataset. Changes of the site radius do not affect REVEALS estimates of taxa with intermediate fall speed of pollen (Cerealia, *Picea*, *Fagus*). The simulation also showed the expected pattern – larger sites have a stronger influence. This effect is again the strongest for taxa with the heaviest and lightest pollen grains. As radii of our sites within each region vary substantially, we could expect high variability between estimates from each leave-one-site-out run. However, taxa with intermediate fall speed of pollen represent dominants of actual regional vegetation, so our results appear to be robust. Even when we omitted one large site from the analysis, the estimates remained significantly similar to those provided by the analysis of all sites in the dataset. The variability of leave-one-site-out out runs for Northern Bohemia showed a pattern that agreed with the simulation. Omitting a large site yielded the most aberrant results.

### Alternative Radius of Sedimentation Basin (R) for Local Taxa

Setting a small radius of the sedimentation basin for local taxa decreases their REVEALS estimates, which would otherwise be too high because of their high pollen proportion. In accordance to this simulation result, the setting had the same effect on real data. Their estimates are even closer to actual vegetation than in the S4MS scenario (Table 3). This possibly confirms our initial hypothesis that the presence of local taxa in the sedimentation basin can be corrected by a smaller radius.

One could object that this technique is too subjective, because the decision as to which pollen is assumed local is made based on the high pollen proportion. We argue that it can be a way of formalizing certain *a priori* information that is well known among palynologists. This knowledge can be obtained from the fossil record either by establishing pollen percentage thresholds or combining pollen and macrofossil data [47].

### Selection of PPEs

PPEs of half of the taxa giving the best match between REVEALS estimates and regional vegetation were determined on the Swiss Plateau [40] (*Pinus*, *Picea*, *Abies* and *Betula*) or in other regions of Central Europe [21], [41], [48] (*Fagus*, Cerealia, *Carpinus*, *Quercus* and *Acer*). PPE values of *Alnus*, *Salix*, *Plantago lanceolata*, *Corylus*, *Ulmus*, *Fraxinus* and *Tilia* originating from northern Europe do not differ substantially (less than two-fold) from values ascertained in Central Europe.

The close semblance between Swiss and Czech PPE data is due to the geographical proximity of the regions under study and the similarity in their climatic conditions, especially when compared with Northern Europe. Apart from the climate, similarities between the landscape mosaics in the Czech and Swiss studies may also play an important role. Both studies deal with plantations of *Pinus* and *Picea*, which create dense and shadowy stands. This explains the lower pollen productivities than those determined in Northern Europe. Moreover, the similarity with the Swiss Plateau also exists on the taxonomic level. Czech and Swiss Cerealia are dominated by autogamous *Triticum* whereas north-European fields host anemogamous *Secale*. Furthermore, we extracted the most suitable PPEs for main vegetation dominants in our study, including both the highest and lowest pollen producers from the study carried out on the Swiss Plateau. PPEs of the remaining taxa (except *Carpinus* and *Ulmus*) are comparable to this data set. This demonstrates the consistency among different PPE studies.

The unexpectedly bad suitability of PPEs from Central Bohemia might be caused by the small size of the sampling area. The PPEs were calculated for an area of 56 km^2^ [21], which can magnify any local anomaly. By contrast, sampling sites on the Swiss Plateau and in southern Sweden are scattered over an area which corresponds to the background pollen area (10^4^–10^5^ km^2^).

### Adjustment of PPEs

Most adjusted PPEs calculating the wind speed setting of 4 m/s remain within the range of standard errors of original values. The PPE of *Ulmus* exceeds this range whereas adjusted PPE values of *Fraxinus*, *Quercus* and *Carpinus* are lower than published values. Such discrepancies might be caused by the scarcity of data (one or two) for *Ulmus* and *Carpinus.* The value for *Quercus* is significantly lower than northern European data, but still reasonably close to Central European data.

A problem arises, however, with adjusted PPEs calculated using the lowered setting of the radius for local taxa (S4MSR_P). The values of these PPEs fall well out of the ranges of original PPEs and seem very improbable. Their relationship to PPEs from pollen/vegetation studies and the ERV model remains unclear. A simulation of PPEs in a heterogeneous landscape with a gradient of local taxa might prove interesting.

## Conclusions

Our simulation shows that changing the site radius influences substantially the REVEALS estimates of taxa with very heavy (*Abies*) or light pollen grains. Decreasing the site radius has a similar effect as increasing the wind speed parameter. We conclude that the initial mismatch between the estimates of *Abies* and its actual proportion was caused by an inappropriate setting of the wind speed parameter. We propose that wind speed should be set to 4 m/s, which is the average wind speed during the flowering season in most regions of the Czech Republic [42].

We found the best set of PPE values and adjusted them to make them as appropriate as possible for estimating present-day vegetation using the REVEALS algorithm. Most PPE values originate either from Central Europe (Swiss Plateau and Central Bohemia) or do not markedly differ from those values (more than two-fold). Ad hoc adjustment of PPEs with respect to present vegetation under the setting of wind speed 4 m/s improves the match 3–4-fold. We consider these values to be appropriate, because all except four of them fall within the ranges of standard errors of original PPEs and retain their relationship with original PPEs. The fact that even adjusted PPEs are cohesive with natural values confirms the theoretical assumption that PPE values from different studies are compatible.

Our initial hypothesis that the effect of local taxa can be corrected by decreasing the radius of the sedimentation basin is correct; however, satisfactory adjustments of PPEs to this setting remain to be identified.

## Supporting Information

Table S1 **List of pollen sites used for REVEALS estimates of present-day vegetation.**
(XLS)

Table S2 **Table of mean values of the cover of Poaceae,** ***Corylus*** **and** ***Plantago lanceolata*** **extrapolated into habitat classes.** Codes of habitats correspond to [36]. Standard total vegetation cover was taken from [34], [35,35]. “Segments total” means all segments of habitats distinguished during the mapping with presence or abundance data. “Average abundance” was calculated from segments with abundance data and presence data.
(XLS)

Table S3 **Table of mean values of the cover of Poaceae,** ***Corylus*** **and** ***Plantago lanceolata*** **extrapolated into CORINE classes.** For further information, see Table S2.
(XLS)

Table S4 **Actual vegetation (%) in each region.** The brackets show standard deviation.
(XLS)

Text S1 **REVEALS model in R-script.**
(DOC)
