# Supplementary material for: Present-Day Vegetation Helps Quantifying Past Land Cover in Selected Regions of the Czech Republic
Source: PLoS One. 2014 Jun 17;9(6):e100117. doi: 10.1371/journal.pone.0100117 (PMC4061133; doi:10.1371/journal.pone.0100117)
Supplement: Text S1 — REVEALS model in R-script. (DOC) [file pone.0100117.s005.doc]

**REVEALS script manual**

REVEALS algorithm is theoretically developed and described in detail by Shinya Sugita (2007). The structure of following script was adopted from his original program. Main aim of the REVEALS migration to R-project environment is possibility of further editing and batch processing.

**Input data:**

The script requires a set of input files as follows:

1. **list of pollen sites**, i.e. list of names of the files from (2), e.g.:

site1.csv

site2.csv

...

1. **pollen data from sites** included in (1) as separated files. First row of this file is a header. It contains names of the time windows. If any site has no record in some time window, column must be included anyway and pollen counts are filled with zeros. Second row contains radiuses of sedimentation basin. Third row lists codes of the deposition model. If pollen was deposited in a bog, use Prentice model (Prentice, 1985) by writing: 1. Any other option, e.g.:2, will process lake model (Sugita, 1993). Fourth and next rows are different taxa and their pollen counts, e.g.:

taxa,0-500BP,500-1000BP,...

Radius,100,100,...

Model,1,2,...

taxon1,0,100,...

taxon2,0,254 ,...

...

1. **pollen productivity estimates and fall speed of pollen**. First row of this file is a header, then follows list of taxa with pollen productivity estimate (first value) and fall speed of pollen (second value). Order of taxa must correspond to ordering in pollen data (2).

taxa,alpha,vg

taxon1,4.2,0.021

taxon2,2.31,0.022

...

All these data files have to be placed in the working directory of R. All these data files must be in formatted as Comma Separated Values (*.csv).

When files are prepared, you start R, select working directory with your files, copy distance weighting function and reveals function into a console. Reveals function has following syntax:

> reveals(file_name_list,file_name_avg,u,Zmax,r)

**parameters**:

1. **file_name_list** - name of the file with the list of pollen sites (1)
2. **file_name_avg** - name of the file with pollen productivity estimates and fall speed of pollen (3)
3. **u** - wind speed (m/s)
4. **Zmax** - radius of the area of background component (km)
5. **r** - radius of the sedimentation basin (m)

Example, create three files from the example (below), copy a REVELAS script code (below) into the R and call by following :

> reveals("my_site_list.csv", "alpha.csv", 3, 60, 100)

**Output** is a table of REVEALS estimates (proportions) for all time window (columns) and all taxa (rows). Output is returned on the screen and also written to the working directory as "results_my_site_list.csv".

**REVEALS script code**

library(zipfR)

### DEFINE Sugita's KP

KPf <- function(vg,u,Zmax,radius,model)

{

b<-75.2*vg/u

if(model=="Prentice")

#Prentice bog model

{

KP<-(exp(-1*b*radius^0.125)-exp(-1*b*(Zmax*1000)^0.125))

}

else

#Sugita lake model

{

xa<-b*(Zmax*1000-radius)^(1/8)

xb<-b*(Zmax*1000+radius)^(1/8)

xc<-b*(radius+radius)^(1/8)

KP<-(4*pi*radius/(b^8))*(Igamma(8,xa)-Igamma(8,xb)+Igamma(8,xc))

}

return(KP)

}

##############################################

reveals <- function(file_name_list,file_name_avg,u,Zmax,r)

{

avg <- read.table(file_name_avg, row.names=1, header=T, sep=",")

lst <- read.table(file_name_list)

paldatasample <- read.table(as.character(lst[1,]),sep=",", row.names=1, header=T)

veg <- matrix(nrow=length(row.names(paldatasample))-2,ncol=length(paldatasample))

rownames(veg) <- rownames(paldatasample)[3:length(rownames(paldatasample))]

colnames(veg) <- colnames(paldatasample)

## LOOP FOR TIMELAYERS

for (w in 1:(length(paldatasample)))

{

## LOOP FOR ALL SITES

allsites <- matrix(nrow=nrow(avg), ncol=length(rownames(lst)))

allsitesprop <- matrix(nrow=nrow(avg), ncol=length(rownames(lst)))

for (m in 1:(length(rownames(lst))))

{

polcount <- read.table(as.character(lst[m,]),sep=",", row.names=1,header=T)

polcount[is.na(polcount)]<-0

## pollen sum

sumv<-0

for (j in 1:(length(rownames(polcount))-2))

{

vg <- avg[j,2]

radius <- r

if(polcount[2,w]==1){model<-"Prentice"

}else{

model<- "Sugita"}

v<-polcount[j+1,w]/(avg[j,1]*KPf(vg,u,Zmax,radius,model))

sumv <- sumv+v

}

## vegetation proportion for 1 species

for (i in 1:(length(rownames(polcount))-2))

{

vg <- avg[i,2]

radius <- r

if(polcount[2,w]==1){model <-"Prentice"

}else{

model <-"Sugita"}

v1 <- polcount[i+2,w]/(avg[i,1]*KPf(vg,u, Zmax,radius,model))

allsitesprop[i,m] <- v1/sumv

allsites[i,m] <- v1

}

} ## END OF ALL SITES LOOP

veg[,w] <- rowSums(allsites)/sum(rowSums(allsites))

} ## END OF TIMELAYERS LOOP

write.table(veg, paste("result_from_",file_name_list, ".csv", sep=""), sep=",", quote=F)

return(veg)

}

**Example files**

Copy text into a empty notepad file with following name. (Do not forget to put one empty line after last line.)

file "my_site_list.csv":

example.csv

file "example.csv":

plants,0

Radius(m),18

Model(Prent_is1Sugit_is2),1

Alnus,100

Salix,100

Fraxinus,100

Betula,100

Corylus,100

Pla_lanc,100

Pinus,100

Ulmus,100

Tilia,100

Quercus,100

Poaceae,100

Carpinus,100

Picea,100

Acer,100

Fagus,100

Cerealia,100

Abies,100

file "alpha.csv":

,alpha,vg

Alnus,4.2000000,0.021

Salix,2.31,0.022

Fraxinus,0.6700000,0.022

Betula,2.4200000,0.024

Corylus,1.4000000,0.025

Pla_lanc,0.9000000,0.029

Pinus,1.3500000,0.031

Ulmus,1.2700000,0.0320000

Tilia,0.8,0.032

Quercus,1.76,0.035

Poaceae,1.0000000,0.035

Carpinus,2.1,0.042

Picea,0.5700000,0.056

Acer,0.3200000,0.056

Fagus,1.2000000,0.057

Cerealia,0.0462000,0.06

Abies,9.92,0.12
